# The combined compartmental maxillary resection: technique codification and preliminary multicenter series analysis

**DOI:** 10.3389/fonc.2026.1908312

**Published:** 2026-08-05

**Authors:** Remo Accorona, Carmine Prizio, Alfred Aga, Naveen Hedne Chandrasekhar, Joseph Williams, Enrico Fazio, Andrea Lorenzi, Francesca Neirotti, Alice Cremasco, Niccolò Mevio, Francesco Pilolli, Andrea Achena, Domenico di Furia, Andrea Figus, Joseph Chun-Kit Chung, Narayanan Prepageran, Alberto Schreiber, Deepa Nair, Narayana Subramaniam, Giovanni Succo, Armando De Virgilio, Jeffery Scott Magnuson, Luca Calabrese, Alberto Giulio Dragonetti, Luca Gazzini

**Affiliations:** 1 Department of Otolaryngology–Head and Neck Surgery, ASST Grande Ospedale Metropolitano Niguarda, Milan, Italy; 2 Department of Otolaryngology, American Hospital I, Tirana, Albania; 3 Department of Head and Neck Surgical Oncology, Apollo Proton Cancer Centre, Chennai, Tamil Nadu, India; 4 Department of Otolaryngology–Head and Neck Surgery, Bolzano Hospital, AS Alto Adige, Bolzano, Italy; 5 Division of Otolaryngology, Department of Surgical Sciences, University of Turin, Turin, Italy; 6 Department of Mechanical and Aerospace Engineering (DIMEAS), Politecnico di Torino, Turin, Italy; 7 Department of Health Sciences, University of Eastern Piedmont “Amedeo Avogadro”, Novara, Italy; 8 Division of Otolaryngology, Department of Health Sciences, University of Milan, Milan, Italy; 9 Division of Plastic Surgery and Microsurgery, Department of Surgical Sciences, University of Cagliari, Cagliari, Italy; 10 Division of Otolaryngology–Head and Neck Surgery, Department of Surgery,University of Hong Kong, Pok Fu Lam, Hong Kong SAR, China; 11 Department of Otolaryngology, Faculty of Medicine, Universiti Malaya, Kuala Lumpur, Malaysia; 12 Department of Otolaryngology–Head and Neck Surgery, Manerbio Hospital, ASST Garda, Manerbio, Italy; 13 Department of Head & Neck Surgery, Tata Memorial Centre, Homi Bhabha National Institute, Mumbai, India; 14 Department of Head and Neck Surgery & Oncology, Aster Hospitals, Bengaluru, Karnataka, India; 15 Department of Oncology, University of Turin, Turin, Italy; 16 Department of Otolaryngology–Head and Neck Surgery, San Giovanni Bosco Hospital, ASL Città di Torino, Turin, Italy; 17 Department of Sense Organs, Sapienza University of Rome, Rome, Italy; 18 Department of Otolaryngology–Head and Neck Surgery, University of Central Florida College of Medicine, Orlando, FL, United States

**Keywords:** combined approach, compartmental surgery, infratemporal fossa, masticator space, maxillary resection, oral cavity cancer, pterygopalatine fossa, resection margins

## Abstract

**Purpose:**

Maxillary cancers extending to the deep craniofacial spaces represent one of the most challenging scenarios in head and neck oncology. Integrating compartmental surgery principles with combined endoscopic–open approaches may optimize deep-margin control, a key determinant of oncologic radicality.

**Methods:**

A retrospective analysis included 34 patients treated between 2019 and 2025 at four referral centers using combined compartmental maxillary resection (CCMR). Procedures were assigned to CCMR types 1–3 according to the compartmental resection performed; preoperative imaging, including assessment of posterior deep spaces and the infratemporal fossa (ITF), informed template selection.

**Results:**

ITF infiltration was absent in 38.2% of cases, partial in 35.3%, and extensive in 26.5%. Resection types were CCMR type 1 in 32.4%, type 2 in 38.2%, and type 3 in 29.4%. Negative margins were achieved in 79.4%; posterior and medial margin control remained reliable even with partial or extensive ITF involvement. Median follow-up was 21 months. Local control rates at one and three years were 81.9% and 75.1%, respectively.

**Conclusion:**

CCMR appears to be a feasible and anatomically standardized surgical strategy for selected maxillary cancers arising from the oral cavity or maxillary sinus with deep craniofacial extension. By integrating compartmental resection principles with combined endoscopic–open approaches, this technique may support margin-oriented surgical planning and reliable control of deep posterior and medial margins, including in cases with ITF involvement. Further studies with longer follow-up are needed to define its oncologic impact.

## 1 Introduction

### 1.1 Clinical background

Maxillary malignancies arising from the upper oral cavity (upper gingivobuccal sulcus or hard palate) and from the maxillary sinus share a common surgical challenge: a marked tendency to extend along posterior craniofacial corridors toward the pterygopalatine fossa (PPF) and infratemporal fossa (ITF), where margin control is technically demanding (1, 2). The upper alveolar ridge and hard palate are in direct continuity with the maxillary sinus. Thin alveolar cortices and frequent pneumatization create low-resistance pathways for tumor spread from the alveolus to the sinus floor and posterior wall. Posteriorly, the maxillary tuberosity lies against the pterygomaxillary fissure, opening into the PPF, a compact neurovascular hub that communicates with the ITF, orbit (via the inferior orbital fissure), nasal cavity (via the sphenopalatine foramen), and middle cranial fossa (via the foramen rotundum). Laterally and posterosuperiorly, the ITF and masticator space contain the pterygoid muscles, maxillary artery and venous plexus, and the maxillary (V_2_) and mandibular (V_3_) divisions of the trigeminal nerve (CN V), providing loose connective tissue and perineural planes that facilitate disease progression.

In both subsites, locoregional failure most often originates at the posterior–medial deep margin, where thin bony partitions and neurovascular pathways facilitate deep soft-tissue and perineural spread and may compromise the integrity of the en bloc specimen (1, 3). These patterns also influence operability. In oral cavity primaries, masticator space involvement is classified as T4b according to the 8th edition of the AJCC staging system and is frequently approached as borderline operable, whereas analogous posterior extension in maxillary sinus tumors may remain resectable in selected patients in the absence of frank skull-base invasion or critical neurovascular encasement (4). Accordingly, outcomes in advanced maxillary cancers are driven less by the staging label than by the topography of deep space invasion and the feasibility of achieving a clear posterior–medial margin with appropriately extended approaches (5, 6).

### 1.2 Surgical strategies for deep-margin control

Two complementary concepts have been proposed to reduce deep-margin failure in advanced maxillary cancers: compartment-based radicality and multiportal access. Compartmental surgery defines radicality anatomically—removing the entire functional unit at risk, including muscular insertions and relevant neurovascular conduits—rather than relying on geometric wide margins. This paradigm has improved locoregional control in advanced oral cavity subsites and has been extended to masticator space disease, where infranotch involvement appears more amenable to compartmental clearance than supranotch spread toward skull-base foramina (7–9).

In parallel, combined endoscopic–open strategies leverage endonasal endoscopy to directly visualize posterior and medial limits within the maxillary sinus and pterygoid region, allowing controlled work at the pterygomaxillary junction and safer skull-base detachment—steps that can be difficult or effectively blind with transfacial exposure alone (10). Contemporary endoscopic-assisted series report high rates of negative posterior margins, supporting multiportal approaches when tumors abut deep corridors, particularly when integrated with compartmental principles for the masticator unit (11).

We describe combined compartmental maxillary resection (CCMR), a standardized technique with three templates (types 1–3) aligned with progression along the PPF/ITF axis. CCMR integrates endoscopic and open corridors to secure posterior–medial margins while preserving an interpretable en bloc specimen.

The rationale of this strategy is to overcome some of the technical limitations of traditional transfacial resections, in which the deep posterior–medial limit is often reached only at the final stages of the procedure and may be difficult to assess under direct visualization. By performing the endoscopic transpterygoid phase first, the surgeon can identify and control the pterygoid and skull-base margins at the beginning of the operation, thereby integrating posterior–medial margin assessment into the initial surgical planning.

The objective of this multicenter study is to provide a preliminary technical codification of this combined approach and to assess its feasibility across high-volume centers. We report margin status and early oncologic outcomes in a cohort at high risk for deep-margin failure, with the aim of exploring whether this standardized, margin-oriented strategy may support curative-intent resection in selected patients with advanced maxillary cancers involving the deep craniofacial compartments.

## 2 Materials and methods

### 2.1 Study design

We conducted a retrospective, multicenter cohort study of consecutive patients selected for CCMR for oral cavity cancer (OCC) or maxillary sinus cancer (MSC). Cases were accrued between January 1, 2019, and December 31, 2025, across four tertiary head and neck oncology centers: ASST Grande Ospedale Metropolitano Niguarda (Milano, Italy; 11 cases), Bolzano Hospital (Bolzano, Italy; 8 cases), American Hospital I (Tirana, Albania; 7 cases), and Apollo Proton Cancer Centre (Chennai, India; 8 cases).

Clinical management was carried out on the basis of accepted oncologic and functional criteria. The study was conducted in compliance with applicable institutional and national ethical requirements and with the principles of the Declaration of Helsinki and its subsequent revisions. According to local regulations and the retrospective nature of the analysis, approval by an ethics committee was not required. Written informed consent for the scientific use of anonymized clinical data had been obtained from all patients.

### 2.2 Patient cohort and eligibility criteria

The analytic cohort comprised 34 adults with maxillary cancer who underwent CCMR as definitive surgical treatment.

Inclusion criteria were: (a) primary tumors of malignant or borderline behavior arising from the upper alveolar ridge, upper gingivobuccal sulcus, hard palate, or maxillary sinus; (b) evidence of posterior maxillary wall involvement and/or tumor extension toward deep craniofacial spaces; and (c) suitability for en bloc maxillary resection with an endoscopic transpterygoid component.

Selected locoregionally persistent or recurrent tumors were also eligible when, following multidisciplinary discussion, a compartmental surgical strategy was considered oncologically advantageous, although these tumors may exhibit a different and potentially more aggressive biological behavior than previously untreated primary tumors. In these cases, surgery was indicated for tumors that had been incompletely resected during previous procedures or had shown an inadequate response to prior chemoradiotherapy. The surgical objective was to remove the entire involved anatomical compartment en bloc, extending the resection beyond the boundaries of any previous surgical procedure.

Exclusion criteria were limited to unresectable disease as determined by the multidisciplinary team and to cases with distant metastases at presentation. Although ITF involvement was classified as T4b disease, it was not considered per se a criterion of unresectability. Tumors were deemed technically unresectable in the presence of radiologically evident skull-base invasion involving the greater wing of the sphenoid and/or the roof of the pterygoid fossa, or encasement of the internal carotid artery or common carotid artery.

All patients underwent contrast-enhanced cross-sectional imaging to define posterior wall erosion, PPF involvement, and the degree of ITF infiltration. ITF involvement was categorized preoperatively as absent, partial, or extensive to guide operative planning and subsequent analyses. Partial involvement was defined as tumor erosion of the posterior wall of the maxillary sinus with extension ≤ 1 cm into the ITF, whereas extensive involvement was defined as tumor extension > 1 cm into the ITF and/or radiologically evident erosion of the pterygoid plates. In cases classified as extensive ITF involvement on CT, contrast-enhanced MRI was performed to better delineate the anatomical structures involved and refine surgical planning.

Cases were reviewed in a multidisciplinary tumor board, and adjuvant therapy was determined postoperatively on the basis of final pathology.

### 2.3 Surgical technique

Combined compartmental maxillary resection is a compartment-oriented, en bloc oncologic resection that combines an endoscopic transnasal transpterygoid phase with an open maxillectomy and, when indicated, compartmental resection of deep craniofacial spaces. The objective is twofold: (1) to achieve precise, visually controlled definition and protection of the deep posterior and medial limits under endoscopic guidance, and (2) to deliver an oncologically radical en bloc specimen via the open phase, minimizing blind maneuvers at critical skull-base interfaces.

#### 2.3.1 CCMR templates

To standardize operative selection and reporting, three CCMR templates (**Table 1**) were codified:

**Table 1:** Combined compartmental maxillary resection (CCMR) clasification.

CCMR type	ITF involvement (imaging)	Endoscopic phase	Open phase	En bloc resection includes
Type 1	No ITF progression; minimal contact at the distal portion of pterygoid plates	Transpterygoid approach; posterior maxillary wall opening ± PPF inclusion	Inferior or subtotal maxillectomy via transoral or midfacial degloving	Inferior or subtotal maxillectomy ± PPF contents
Type 2	Partial ITF infiltration across the posterior maxillary wall	Superior transpterygoid approach; V_2_ and nerve of the pterygoid canal sampling ± frozen section	Coronoidectomy; pterygoid muscles detachment from mandible and skull base with transfacial approach	Subtotal or radical extended maxillectomy with PPF, pterygoid, ITF contents
Type 3	Extensive ITF infiltration	Analogous to type 2	Masticator-space compartmental resection with mandibular ramus with transfacial approach ± temporal counter-incision	Subtotal to radical extended maxillectomy with masticatory space resection

ITF, infratemporal fossa; PPF, pterygopalatine fossa.

**Figure 1:**
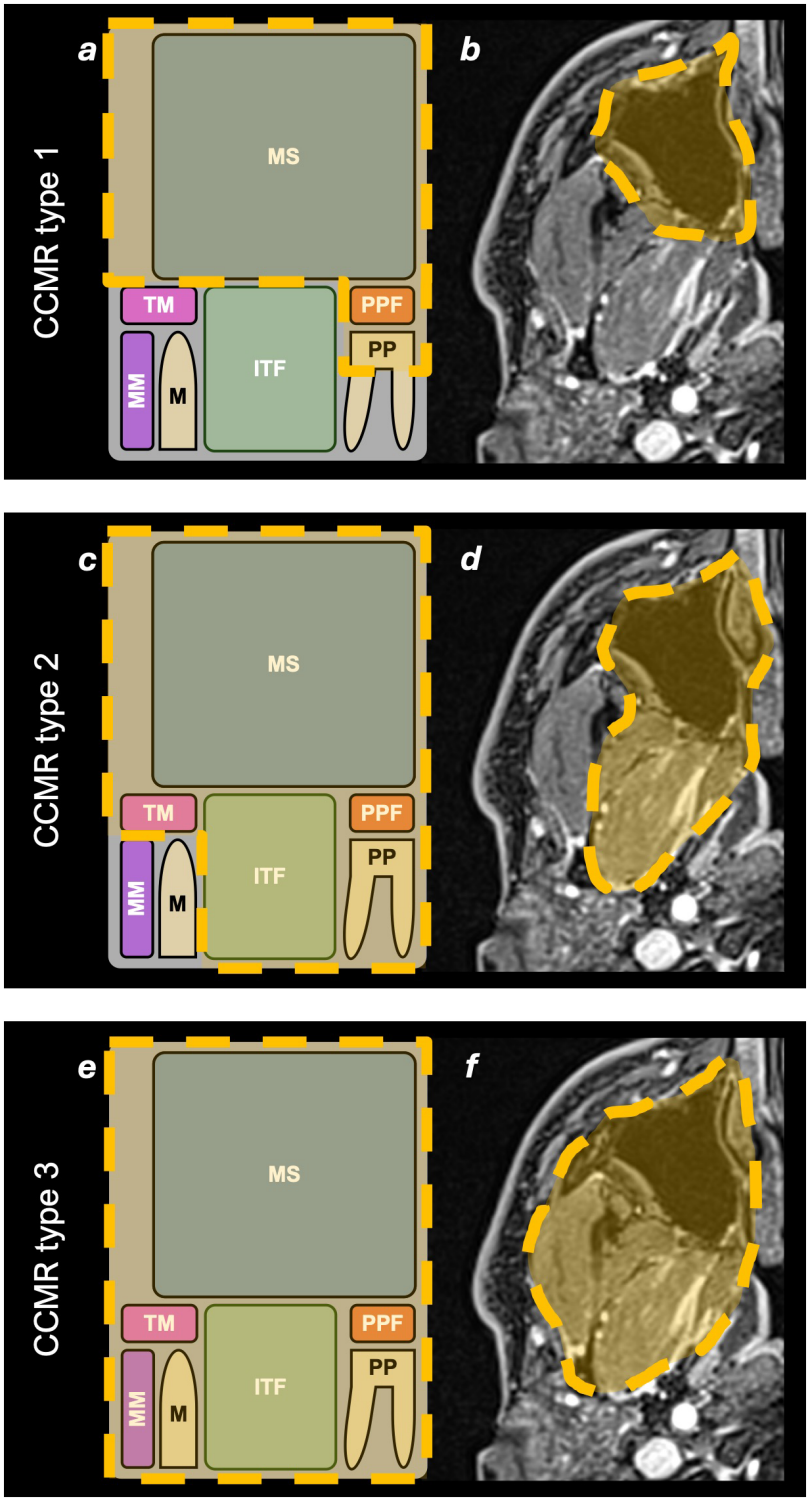
Proposed combined compartmental maxillary resection (CCMR). **(a, b)** CCMR type 1; **(c, d)** CCMR type 2; **(e, f)** CCMR type 3. ITF, infratemporal fossa; M, mandible; MM, masseter muscle; MS, maxillary sinus; PP, pterygoid process; PPF, pterygopalatine fossa; TM, temporalis muscle.

- CCMR type 1 (no radiologic ITF progression; minimal contact at the distal portion of pterygoid plates)—The endoscopic phase includes sectioning of the inferior pterygoid plate and opening of the posterior maxillary wall for endoscopic ligation of the maxillary artery; en bloc inclusion of PPF contents is optional but often required. Open completion comprises an inferior or subtotal maxillectomy via a transoral approach or midfacial degloving (**Figures 1a, b**).
- CCMR type 2 (partial ITF infiltration through the posterior maxillary wall)—A transpterygoid endoscopic phase addresses the basisphenoid, with identification and management of the nerve of the pterygoid canal and V_2_. The open phase includes coronoidectomy to access the ITF and complete detachment of the pterygoid musculature from mandibular and skull-base insertions. En bloc removal encompasses a subtotal-to-radical maxillectomy with compartmental resection of PPF and ITF contents (**Figures 1c, d**).
- CCMR type 3 (extensive ITF and masticator space involvement)—The endoscopic phase mirrors type 2 and is combined with an expanded open compartmental resection of the masticator space, including sacrifice of the mandibular ramus. Maxillectomy ranges from subtotal to radical extended, ensuring full compartmental clearance of the masticator space. A temporal counter-incision can be added as needed to compartmentalize the temporalis insertion when the tumor extends beyond the sigmoid notch (**Figures 1e, f**).

Across templates, the endoscopic stage prioritized definition of the deep margin; the open stage then delivered the specimen en bloc along pre-established planes, avoiding piecemeal disassembly of at-risk corridors.

#### 2.3.2 Endoscopic phase

Under image-guided endoscopic sinus surgery, a tailored transpterygoid approach was performed according to tumor extent. After creation of a standard sinonasal corridor (middle meatal antrostomy with posterior wall access as required), the posterior maxillary wall was opened to expose the PPF. Dissection proceeded to identify and control the terminal branches of the maxillary artery within the PPF. The contents of the PPF were resected en bloc with the specimen when included in the template.

**Figure 2:**
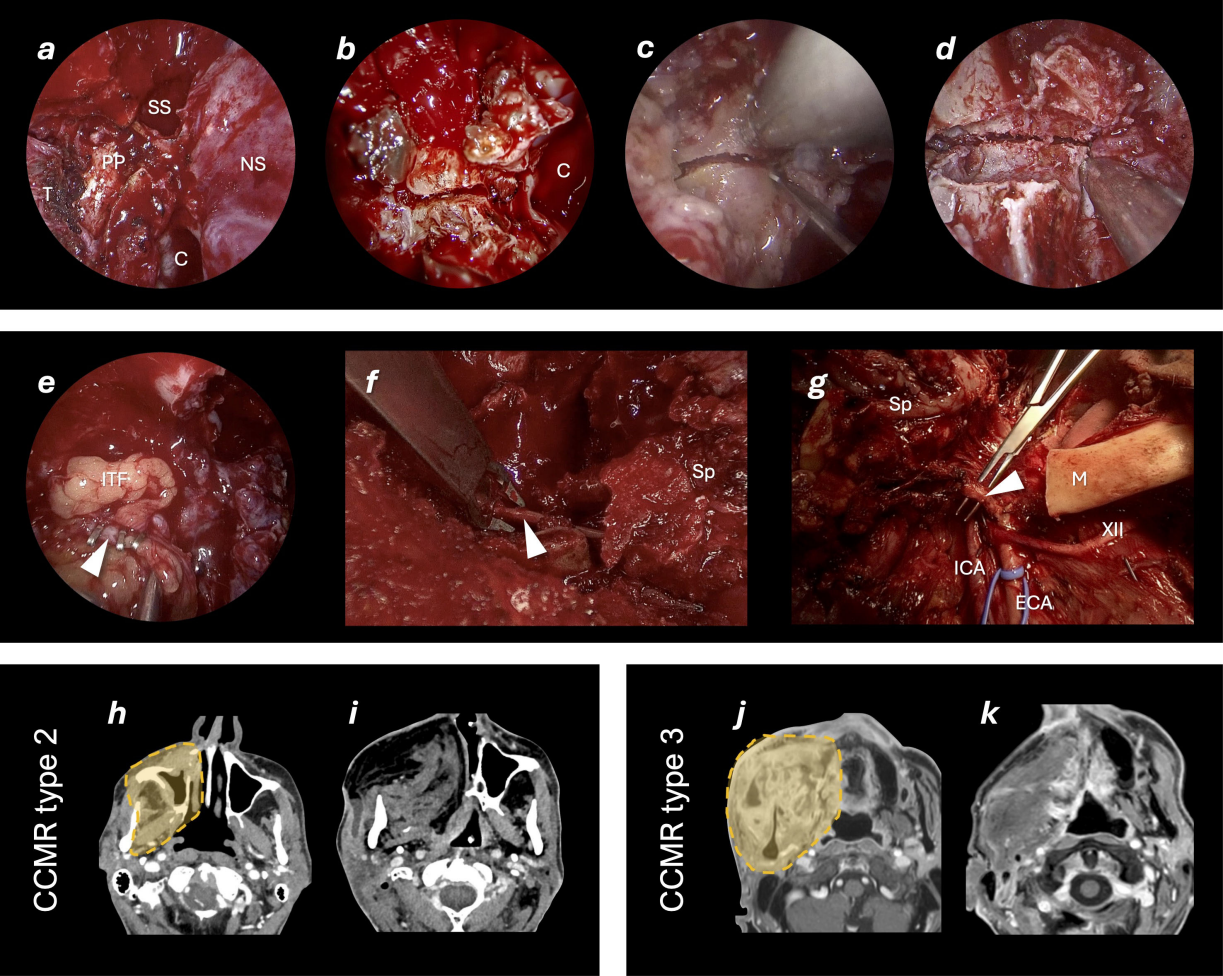
Summary of key surgical steps and postoperative imaging after CCMR. Endoscopic pterygoid management (first row): **(a)** Exposure of the pterygoid plates; **(b)** example of inferior pterygoid osteotomy to facilitate maxillary detachment in CCMR type 1; **(c)** linear osteotomy using piezosurgery followed by **(d)** completion with a drill. Vascular control (second row): (**e)** Endoscopic endonasal ligation of maxillary artery (arrow) in CCMR type 1; **(f)** open transfacial ligation of maxillary artery (arrow) after coronoidectomy and maxillary mobilization in CCMR type 2; **(g)** open neck ligation of the external carotid artery (arrow) after segmental mandibulectomy in CCMR type 3.=Representative outcomes after CCMR types 2 and 3 (third row): **(h)** Preoperative CT showing a tumor with partial ITF involvement, consistent with the indication for CCMR type 2; **(i)** postoperative CT of the same patient showing complete ITF clearance, reconstruction with an ALTFF, and mandibular coronoidectomy. **(j)** Preoperative MRI showing a tumor with extensive ITF invasion, consistent with the indication for CCMR type 3; **(k)** postoperative MRI of the same patient showing complete clearance of the masticator space, reconstruction with an ALTFF, and segmental mandibulectomy C, choana; ECA, external carotid artery; ICA, internal carotid artery; ITF, infratemporal fossa; M, mandible; NS, nasal septum; PP, pterygoid plates; Sp, specimen; SS, sphenoid sinus; T, tumor; XII, hypoglossal nerve.

The pterygoid process was then exposed, drilled, and sectioned. In more advanced cases, a higher transpterygoid route was fashioned to address the basisphenoid; the nerve of the pterygoid canal and V_2_ were identified and managed to secure the posterior and medial limits, eventually tested with proximal frozen section to evaluate extension through the skull base. This endoscopic step was designed to convert critical deep margins into well-visualized planes before the open en bloc detachment (**Figures 2a–d**).

#### 2.3.3 Open phase

The open phase consisted of a transoral exposure, extended to a transfacial approach when required. Transfacial access was achieved using a Moure lateral rhinotomy, midfacial degloving, or a Weber–Fergusson incision, with adjunct extensions as needed (e.g., Dieffenbach subciliary modification, Borle extension, Lynch incision, or Longmire incision) (12).

When required to achieve compartmental clearance of the infratemporal and masticator spaces, the open phase included coronoidectomy with complete detachment of the pterygoid musculature (CCMR type 2) and, in more advanced cases, mandibulectomy with sacrifice of the mandibular ramus (CCMR type 3).

#### 2.3.4 Vascular control

Vascular control followed a stepwise escalation:

- purely endoscopic maxillary artery control in CCMR type 1 (**Figure 2e**);
- intra-ITF control after coronoidectomy and maxillary mobilization in CCMR type 2 (**Figure 2f**); and
- systematic control of the external carotid artery distal to the origin of the facial artery in CCMR type 3 (**Figure 2g**).

#### 2.3.5 Extent of maxillectomy

The extent of maxillectomy was selected according to tumor spread, as follows:

- inferior maxillectomy: removal of the segment below an axial plane passing through the infraorbital foramen (typical in CCMR type 1);
- subtotal maxillectomy: superior osteotomy between the infraorbital foramen and the orbital floor (CCMR types 1–3 as indicated);
- total maxillectomy: inclusion of the orbital floor, with extension to the periorbita or extraconal fat when required (usually CCMR types 2–3); and
- radical extended maxillectomy: inclusion of orbital exenteration and/or wide skin resection and/or nasal resection in selected cases (CCMR types 2–3).

#### 2.3.6 Resection margins and compartmental vectors

Resection margins were defined along cardinal anatomical vectors to achieve compartmental clearance (**Figures 2h–k**) (13):

- posterior: towards the upper parapharyngeal space and, if needed, to the skull base at the foramina rotundum, ovale, and spinosum;
- lateral: up to the parotid compartment and overlying skin when infiltrated; and
- medial: extending to the soft palate and, in advanced cases, the nasopharyngeal wall.

### 2.4 Perioperative and postoperative management

Tracheostomy was performed selectively based on airway risk. Reconstruction strategy (prosthetic obturator vs. regional or free tissue transfer) was individualized according to maxillectomy level, soft-tissue deficit, adjuvant therapy plans, and patient performance status.

Postoperatively, patients were monitored for early and late complications. All cases were re-discussed at the multidisciplinary tumor board after final histopathology to determine adjuvant treatment: RT alone (photon beam or, where appropriate, heavy-ion particle therapy), proton beam therapy (PBT), systemic chemotherapy (CTX), or concurrent chemoradiotherapy (CCRT). Management of recurrence followed tumor board consensus and included salvage surgery, systemic therapy, or best supportive care.

### 2.5 Data collection and statistical analysis

Clinical, radiologic, operative, and pathologic variables were abstracted from prospectively maintained institutional surgical oncology registries and harmonized across centers using a shared codebook. Collected data included demographics, tumor subsite, preoperative imaging features (including ITF category), CCMR template, extent of resection and reconstruction, perioperative course and complications, and postoperative treatments. Clinical and pathological staging (cTNM and pTNM) was assigned according to the 8th edition of the AJCC staging system, and key pathologic variables (including margin status, perineural and lymphovascular invasion, and nodal status) were recorded from final reports.

Continuous variables are summarized as mean ± standard deviation (SD), range, and median with interquartile range (IQR). Categorical variables are presented as counts and percentages.

Time-to-event endpoints were calculated from the date of index CCMR surgery (time zero). Overall survival (OS) was defined as time from surgery to death from any cause or the last follow-up. Disease-specific survival (DSS) was defined as time from surgery to death attributable to the index cancer or the last follow-up. Disease-free survival (DFS) was defined as time from surgery to first evidence of recurrence (local, regional, or distant) or the last follow-up. Local control (LC) was defined as the time from surgery to the first evidence of persistent or recurrent disease at the primary tumor site or to the last follow-up.

OS, DSS, DFS, and LC were analyzed using the Kaplan–Meier method. Survival curves were compared using the log-rank (LR) test and the Gehan–Breslow–Wilcoxon (GBW) test. All tests were two-sided with a significance threshold of *p* < 0.05.

Statistical analyses were performed in RStudio, version 2026.01.0 (RStudio PBC, Boston, MA).

## 3 Results

### 3.1 Patient characteristics

Thirty-four patients treated across four tertiary oncology centers met inclusion criteria and underwent CCMR for OCCs and MSCs (**Table 2**). The cohort included 23 men (67.6%) and 11 women (32.4%) (M:F ratio, 2.1:1). Mean age at diagnosis was 61.4 ± 17.0 years (range, 21–86; median 64, IQR 54–74). The Charlson comorbidity index showed a median value of 2 (IQR 1–3). Common comorbidities included diabetes mellitus, chronic pulmonary disease, chronic liver disease, cardiovascular disease, and chronic kidney disease, alongside prevalent tobacco smoking and alcohol consumption. Baseline clinicopathologic characteristics are summarized in **Tables 2**, **3**.

**Table 2:** Clinicopathologic characteristics of the 34 patients.

Characteristic		OCCs (n = 24)	MSCs (n = 10)	Total (n = 34)
Age (y)	Mean ± SD	63.8 ± 17.4	55.7 ± 15.5	61.4 ± 17.0
	Median (IQR)	67 (56–75)	58 (45–67)	64 (54–74)
Sex	Male	16 (66.7%)	7 (70.0%)	23 (67.6%)
	Female	8 (33.3%)	3 (30.0%)	11 (32.4%)
CCI	Median (IQR)	3 (1–3)	2 (0–3)	2 (1–3)
Histology	SCC	15 (62.5%)	4 (40.0%)	19 (55.9%)
	MSGC	8 (33.3%)	2 (20.0%)	10 (29.4%)
	Other	1 (4.2%)	4 (40.0%)	5 (14.7%)
Subsite	Upper alveolar ridge	13 (54.2%)	N/A	13 (38.2%)
	Posterior wall of the maxillary sinus	N/A	10 (100%)	10 (29.4%)
	Hard palate	8 (33.3%)	N/A	8 (23.5%)
	Retromolar trigone	2 (8.3%)	N/A	2 (5.9%)
	Buccal mucosa	1 (4.2%)	N/A	1 (2.9%)
Disease status	Primary	17 (70.8%)	4 (40.0%)	21 (61.8%)
	Persistent	3 (12.5%)	4 (40.0%)	7 (20.6%)
	Recurrent	4 (16.7%)	2 (20.0%)	6 (17.6%)
ITF invasion	No	10 (41.7%)	3 (30.0%)	13 (38.2%)
	Partial	9 (37.5%)	3 (30.0%)	12 (35.3%)
	Extensive	5 (20.8%)	4 (40.0%)	9 (26.5%)
CCMR type	Type 1	9 (37.5%)	2 (20.0%)	11 (32.4%)
	Type 2	9 (37.5%)	4 (40.0%)	13 (38.2%)
	Type 3	6 (25.0%)	4 (40.0%)	10 (29.4%)
cT	cT3	5 (20.8%)	0 (0%)	5 (14.7%)
	cT4a	11 (45.8%)	7 (70.0%)	18 (52.9%)
	cT4b	8 (33.3%)	3 (30.0%)	11 (32.4%)
cN	cN0	19 (79.2%)	9 (90.0%)	28 (82.4%)
	cN1	2 (8.3%)	0 (0%)	2 (5.9%)
	cN2	3 (12.5%)	1 (10.0%)	4 (11.8%)
pT	pT2	3 (12.5%)	0 (0%)	3 (8.8%)
	pT3	2 (8.3%)	2 (20.0%)	4 (11.8%)
	pT4a	11 (45.8%)	4 (40.0%)	15 (44.1%)
	pT4b	8 (33.3%)	4 (40.0%)	12 (35.2%)
pN	pNX	8 (33.3%)	1 (10.0%)	9 (26.5%)
	pN0	12 (50.0%)	6 (60.0%)	18 (52.9%)
	pN1	2 (8.3%)	1 (10.0%)	3 (8.8%)
	pN2	1 (4.2%)	2 (20.0%)	3 (8.8%)
	pN3	1 (4.2%)	0 (0%)	1 (2.9%)
Margin status	Negative	19 (79.2%)	8 (80.0%)	27 (79.4%)
	Close	1 (4.2%)	0 (0%)	1 (2.9%)
	Positive	4 (16.7%)	2 (20.0%)	6 (17.6%)
Adjuvant therapy	No	7 (29.2%)	2 (20.0%)	9 (26.5%)
	RT	9 (37.5%)	2 (20.0%)	11 (32.4%)
	CTX	1 (4.2%)	0 (0%)	1 (2.9%)
	CCRT	5 (20.8%)	2 (20.0%)	7 (20.6%)
	PBT	2 (8.3%)	3 (30.0%)	5 (14.7%)
	IO	0 (0%)	1 (10.0%)	1 (2.9%)
Follow-up (mo)	Mean ± SD	25.2 ± 23.3	23.4 ± 17.0	24.8 ± 21.8
	Median (IQR)	20 (8–33)	23 (14–27)	21 (8–32)
Recurrence	No	15 (62.5%)	7 (70.0%)	22 (64.7%)
	Yes	9 (37.5%)	3 (30.0%)	12 (35.3%)

CCI, Charlson comorbidity index; CCMR, combined compartmental maxillary resection; CCRT, chemoradiotherapy; CTX, chemotherapy; IO, immunotherapy; IQR, interquartile range; ITF, infratemporal fossa; MSCs, maxillary sinus cancers; MSGC, malignant salivary gland carcinoma; N/A, not applicable; OCCs, oral cavity cancers; PBT, proton beam therapy; RT, radiotherapy; SCC, squamous cell carcinoma; SD, standard deviation.

**Table 3:** Study population: characteristics, treatment, and follow-up.

No.	Sex, Age (y)	Subsite	Disease status	Previous treatment	cTNM	ITF invasion	CCMR type	Maxillectomy	Histology	Margin status	pTNM	Adjuvant treatment	Current status	Recurrence
1	M, 63	UAR	Pri	No	T4bN0	Extensive	3	RE	SCC	−	T4bN0	RT (×)	DOD	Yes (N)
2	M, 76	UAR	Per	RT	T4bN0	Partial	2	Rad	SCC	−	T4bN0	No	DOC	No
3	F, 69	UAR	Pri	No	T4bN0	Partial	2	ST	SCC	−	T4aN1	RT	DOD	Yes (T)
4	M, 71	PWMS	Pri	No	T4bN0	Extensive	3	RE	SCC	−	T4bN0	RT	NED	No
5	M, 73	HP	Pri	No	T4aN0	Absent	1	Inf	SCC	−	T4aNX	RT	NED	No
6	M, 86	UAR	Pri	No	T4aN0	Absent	1	Inf	SCC	≈	T4aN0	RT (×)	NED	No
7	M, 66	PWMS	Pri	No	T4aN0	Absent	1	Rad	MECa	−	T4aN0	RT	NED	No
8	M, 75	UAR	Pri	No	T4aN2c	Partial	2	Rad	SCC	−	T4aN3b	CCRT	NED	No
9	M, 62	HP	Pri	No	T4aN0	Absent	1	Inf	AdCC	−	T4aN1	RT	NED	No
10	M, 67	PWMS	Per	S	T4bN0	Extensive	3	ST	AB	−	T4bNX	No	NED	No
11	F, 58	RMT	Pri	No	T3N0	Absent	1	Inf	AdCC	−	T3NX	RT	NED	No
12	M, 44	PWMS	Per	S + CCRT	T4aN0	Absent	1	Rad	SCC	−	T4aN0	CCRT	NED	No
13	F, 21	UAR	Pri	No	T4aN0	Absent	1	Inf	AdCC	−	T4aN0	PBT	NED	No
14	M, 58	BM	Rec	S + CCRT	T3N0	Partial	3	Rad	SCC	−	T2NX	CTX	DOD	Yes (T)
15	F, 61	PWMS	Pri	No	T4bN0	Partial	2	ST	AdCC	+	T4bN0	PBT	NED	No
16	M, 54	PWMS	Rec	NACT + S	T4aN2b	Extensive	3	Rad	SCC	−	T4aN2b	CCRT	AWD	Yes (T)
17	M, 85	HP	Pri	No	T3N0	Absent	1	ST	SCC	−	T2N0	No	AWD	Yes (T)
18	F, 45	UAR	Pri	No	T3N1	Absent	1	ST	SCC	−	T3N0	CCRT	NED	No
19	M, 42	PWMS	Per	S + CTX	T4aN0	Partial	2	ST	SCC	−	T3N0	PBT	NED	No
20	F, 38	HP	Per	S	T4bN0	Extensive	3	Rad	AdCC	+	T4bN0	RT	DOD	Yes (T)
21	F, 28	UAR	Per	S	T4aN0	Partial	2	ST	OSa	+	T4aN0	CCRT	NED	No
22	M, 80	HP	Pri	No	T4bN0	Partial	2	ST	AdCC	−	T4aNX	RT	AWD	Yes (T)
23	M, 73	HP	Pri	No	T4bN0	Extensive	3	Rad	AdCC	+	T4bNX	RT (×)	DOD	Yes (T)
24	M, 65	HP	Pri	No	T3N0	Absent	1	Inf	MEC	−	T2NX	No	NED	No
25	F, 79	UAR	Pri	No	T4aN0	Absent	2	ST	SCC	−	T4aN0	RT	NED	No
26	M, 56	UAR	Rec	S	T4aN0	Extensive	3	RE	SCC	−	T4bN0	CCRT	NED	No
27	F, 56	UAR	Rec	S	T4aN0	Partial	2	Inf	AdCC	+	T4bNX	PBT	Lost	Yes (M)
28	M, 48	PWMS	Pri	No	T4aN0	Partial	2	Rad	MM	+	T4bN1	PBT	DOD	Yes (N+M)
29	F, 27	PWMS	Per	S + CCRT	T4aN0	Extensive	3	Rad	NUT	−	T3N0	No	DOC	No
30	M, 86	UAR	Pri	No	T4bN1	Extensive	3	Inf	SCC	−	T4bN2b	CCRT	DOD	Yes (T+N)
31	M, 70	RMT	Pri	No	T4bN2b	Partial	2	Inf	SCC	−	T4bN0	RT (×)	NED	No
32	F, 77	PWMS	Rec	S + RT	T4aN0	Absent	2	ST	MM	−	T4aN2b	IO	Lost	Yes (M)
33	M, 74	HP	Rec	S + RT	T4aN0	Absent	1	ST	SCC	−	T4aNX	RT	NED	No
34	M, 55	UAR	Pri	No	T4aN2b	Partial	2	ST	SCC	−	T4aN0	RT	NED	No

+, positive; ≈, close; −, negative; ×, interrupted/refused/not started; AB, ameloblastoma; AdCC, adenoid cystic carcinoma; AWD, alive with disease; BM, buccal mucosa; CCMR, combined compartmental maxillary resection; CCRT, chemoradiotherapy; CTX, chemotherapy; DOC, died of other causes; DOD, died of disease; HP, hard palate; Inf, inferior; IO, immunotherapy; ITF, infratemporal fossa; Lost, lost to follow-up; MEC, mucoepidermoid carcinoma; MECa, myoepithelial carcinoma; MM, mucosal melanoma; NACT, neoadjuvant chemotherapy; NED, no evidence of locoregional disease; OSa, osteosarcoma; PBT, proton beam therapy; Per, persistent; Pri, primary; PWMS, posterior wall of the maxillary sinus; Rad, radical; RE, radical extended; Rec, recurrent; RMT, retromolar trigone; RT, radiotherapy; S, surgery; SCC, squamous cell carcinoma; ST, subtotal; UAR, upper alveolar ridge.

Preoperative imaging demonstrated absent, partial, or extensive ITF involvement in 13 (38.2%), 12 (35.3%), and 9 (26.5%) patients, respectively.

Clinical T categories were cT3 in 5 patients (14.7%), cT4a in 18 (52.9%), and cT4b in 11 (32.4%). All patients were staged as cM0.

Among patients with cT4b OCC (n = 8), five tumors were classified as supranotch and three as infranotch according to Liao’s classification (14).

### 3.2 Operative details

CCMR type 1, type 2, and type 3 resections were performed in 11 (32.4%), 13 (38.2%), and 10 (29.4%) patients, respectively.

The management of the cN0 neck followed a patient-specific approach. Concurrent neck dissection was undertaken in 25 patients (73.5%); the remaining 9 patients (all cN0) did not undergo neck surgery. Of these, four patients had previously received oncological treatments of the neck, while in five cases neck surgery was omitted based on histology or low-risk tumor subsite.

Three patients underwent combined resection involving the orbit, parotid gland, and overlying skin; three required additional parotidectomy; and one underwent additional skin resection.

Reconstruction was tailored to the defect size, the CCMR type, and the patient’s functional requirements. Free tissue transfer was performed in 16 patients (47.1%), utilizing anterolateral thigh flaps (ALTFF; n = 9) and fibula free flaps (FFF) (n = 8); one patient received a combined FFF–ALTFF reconstruction. Regional pedicled flaps were the primary reconstructive choice in 10 patients (29.4%), specifically the temporalis muscle flap (n = 7) and the buccal fat pad (n = 3). The remaining patients were managed with local flaps (n = 5) or prosthetic obturators (n = 3).

**Figure 3:**
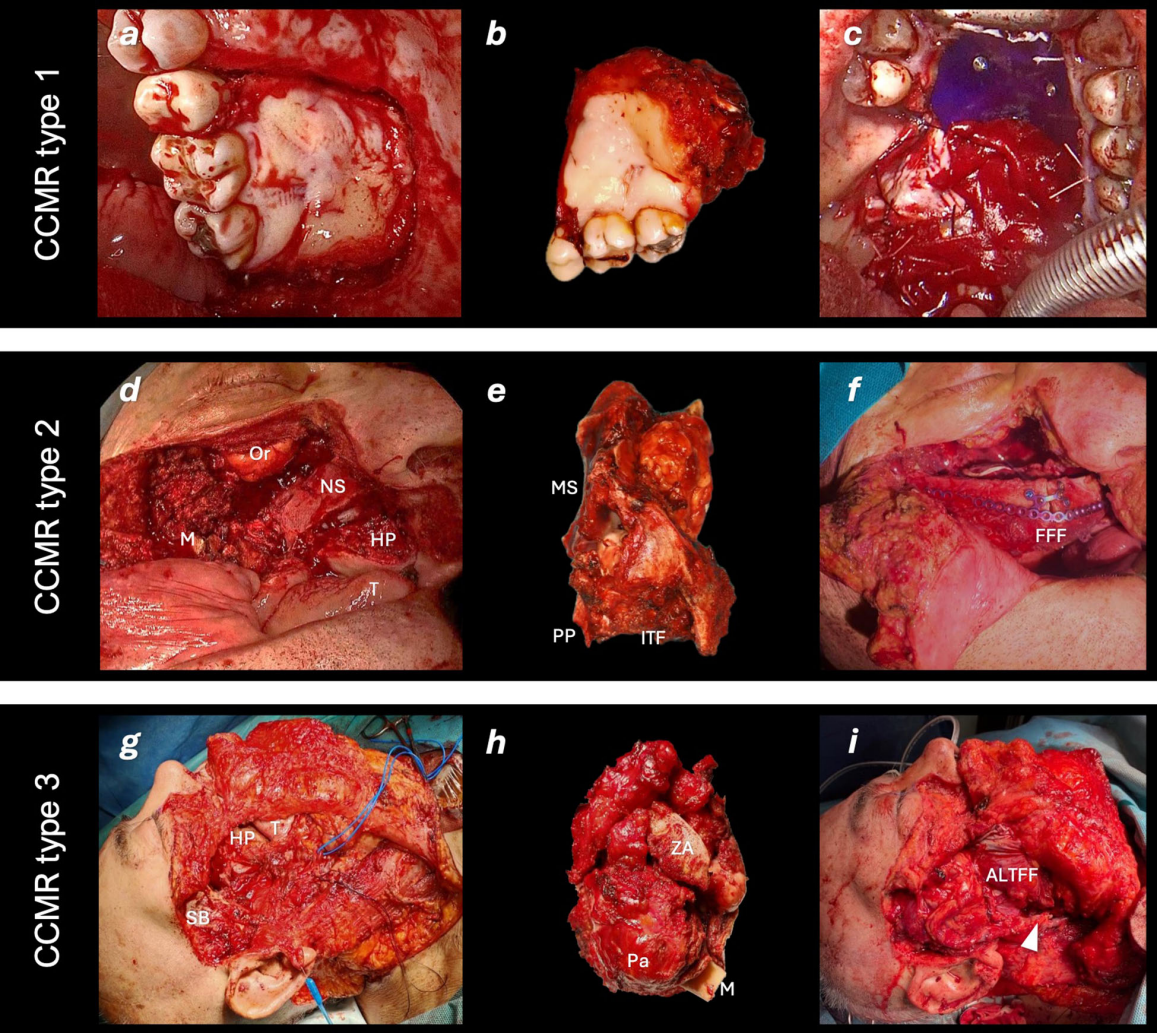
Representative examples of CCMR patterns in the study cohort. CCMR type 1: **(a)** Intraoperative endoscopic view of a patient with oral cavity AdCC focally involving the right hard palate (pT3); **(b)** surgical specimen; **(c)** postoperative endoscopic view showing reconstruction with a pedicled local flap, hard palate prosthesis and synthetic material. CCMR type 2: **(d)** Intraoperative view at completion of the ablative phase of a patient with SCC of the left hard palate with pterygoid plate invasion (pT4a); **(e)** surgical specimen; **(f)** final surgical outcome following reconstruction with a fibula free flap. CCMR type 3: **(g)** Intraoperative view at completion of the ablative phase of a patient with ameloblastoma of the right maxillary sinus with extensive infiltration of the ITF and masticator space; **(h)** surgical specimen; **(i)** final surgical outcome following reconstruction with a chimeric ALTFF with the skin paddle overturned in the oral cavity, the muscular component filling the ITF cranially, and the vascular pedicle (arrow) externally with neck anastomosis. ALTFF, anterolateral thigh free flap; FFF, fibula free flap; HP, hard palate; ITF, infratemporal fossa; M, mandible; MS, maxillary sinus; NS, nasal septum; Or, orbit; Pa, parotid gland; PP, pterygoid process; SB, skull base; T, tongue; ZA, zygomatic arch.

Representative cases for each CCMR type are shown in **Figure 3**.

### 3.3 Pathologic findings

Final margin status was negative (≥ 5 mm) in 27 patients (79.4%), close (< 5 mm) in 1 (2.9%), and positive (ink on tumor) in 6 (17.6%). Detailed analysis of the 7 cases with positive or close margins showed that in 71.4% (n = 5), the tumor reached the soft-tissue limits of the ITF or masticator space at the very edge of the resection, precluding a clear safe margin due to the intrinsic anatomical boundaries of the compartment. In the remaining 28.6% (n = 2), the positive margins were superficial or mucosal, specifically involving the ITF in CCMR type 2 and the masticator space in CCMR type 3.

PNI was present in 9 patients (26.5%), LVI in 6 (17.6%), and bone involvement in 25 (73.5%).

Histology comprised squamous cell carcinoma (SCC) in 19 patients, malignant salivary gland carcinomas in 10 (8 adenoid cystic carcinomas, 1 mucoepidermoid carcinoma, and 1 myoepithelial carcinoma), and other histologies in 5 (1 osteosarcoma, 2 mucosal melanomas, 1 ameloblastoma, and 1 NUT carcinoma).

Pathological T categories were pT2 in 3 patients (8.8%), pT3 in 4 (11.8%), pT4a in 15 (44.1%), and pT4b in 12 (35.2%).

### 3.4 Postoperative course and adjuvant therapy

Median length of stay was 12 days (IQR 6–24). At discharge, 23 (67.6%) patients were taking an oral diet; 6 (17.6%) required a nasogastric tube; and 5 (14.7%) had a percutaneous endoscopic gastrostomy (two of whom were also partially taking oral intake). One patient was discharged with a tracheostomy.

Adjuvant therapy was administered to 25 patients (73.5%). Nine patients (26.5%) did not receive adjuvant treatment; among these, 1 (2.9%) had previously undergone RT, 1 (2.9%) had previously received CCRT, 1 (2.9%) did not start the planned CCRT because of early recurrence, 1 (2.9%) declined the recommended adjuvant therapy, and 2 (5.9%) discontinued RT. In the remaining 3 patients (8.8%), adjuvant treatment was not indicated on the basis of final pathology, including 1 ameloblastoma case, 1 SCC staged as pT2N0, and 1 SCC staged as pT2 cN0.

Median follow-up was 21 months (IQR 8–32).

Disease recurrence occurred in 12 patients (35.3%) during follow-up. Recurrences were predominantly local (7/34; 20.6%), with isolated regional (nodal) relapse in one patient (2.9%) and isolated distant metastasis in two patients (5.9%). Concurrent multi-site relapse was observed in two patients (5.9%), comprising one local plus nodal relapse and one nodal plus distant relapse.

### 3.5 Survival outcomes

Survival analyses were performed in 30 of 34 patients, after excluding ameloblastoma (n = 1), NUT carcinoma (n = 1), and mucosal melanoma (n = 2) cases due to non-comparable tumor biology and prognosis.

**Figure 4:**
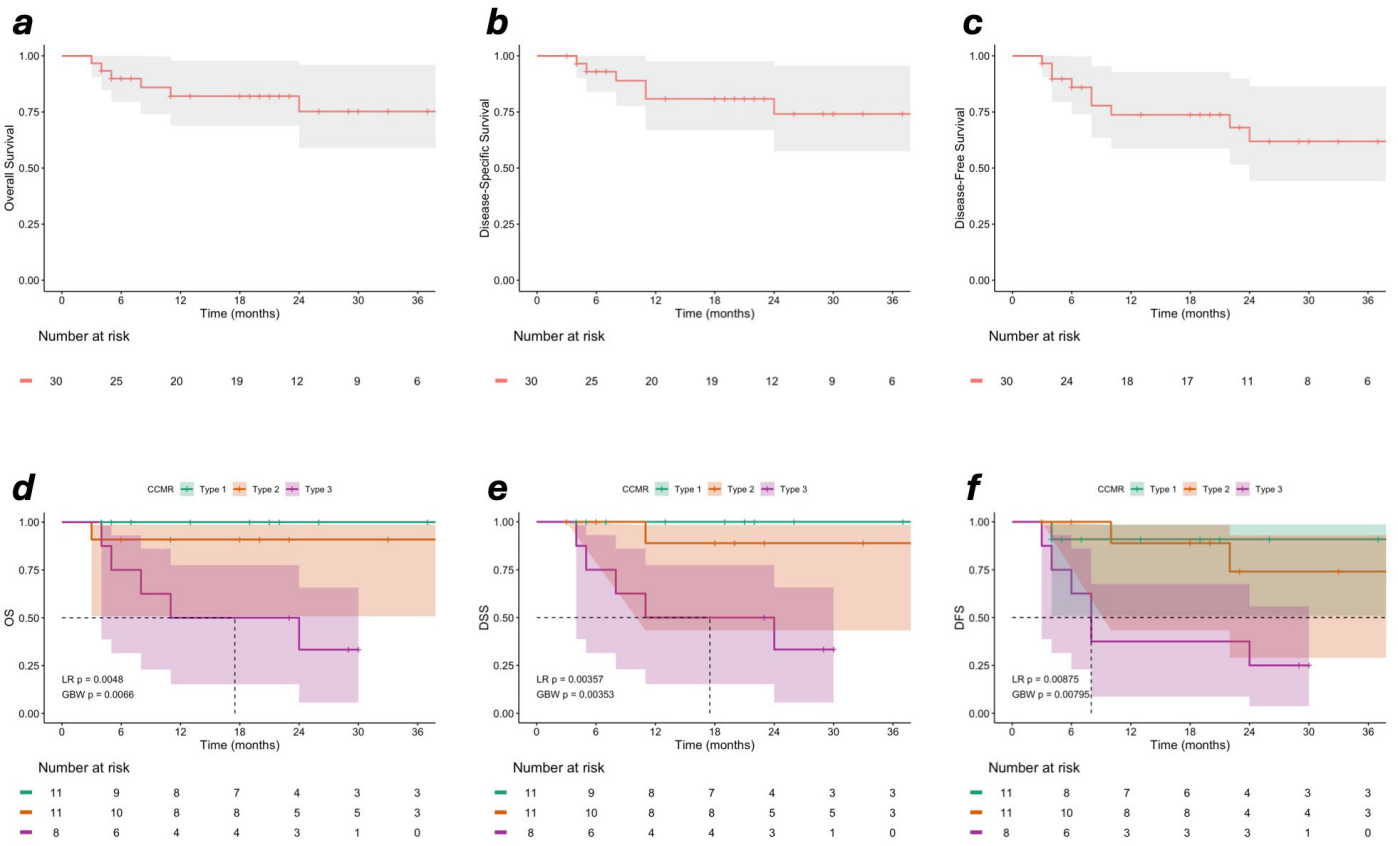
Overall survival (OS, **(a)**, disease-specific survival (DSS,**( b)**, and disease-free survival (DFS, **(c)** in 30 of 34 patients, after exclusion of ameloblastoma, NUT carcinoma and mucosal melanoma. OS **(d)**, DSS **(e)**, and DFS **(f)** by CCMR type.

OS at one and three years was 82.1% (95% CI 68.9–97.7%) and 75.2% (95% CI 58.9–96.0%), respectively. DSS at one and three years was 80.8% (95% CI 67.0–97.5%) and 74.1% (95% CI 57.5–95.5%), respectively. DFS at one and three years was 73.7% (95% CI 58.6–92.7%) and 61.9% (95% CI 44.3–86.5%), respectively (**Figures 4a–c**).

OS for patients undergoing CCMR type 1 (11/30) was 100% at both one and three years (95% CI not estimable, due to absence of events); for CCMR type 2 (11/30), 90.9% at one and three years (95% CI 50.8–98.7%); and for CCMR type 3 (8/30), 50.0% at one year (95% CI 15.2–77.5%) and 33.3% at two years (95% CI 5.6–65.8%), with no three-year estimates available for this group, as follow-up did not extend to three years. DSS for CCMR type 1 was 100% at one and three years (95% CI not estimable); for CCMR type 2, 88.9% at one and three years (95% CI 43.3–98.4%); and for CCMR type 3, 50.0% at one year (95% CI 15.2–77.5%) and 33.3% at two years (95% CI 5.6–65.8%). DFS for CCMR type 1 was 90.9% at both one and three years (95% CI 50.8–98.7%); for CCMR type 2, 88.9% at one year (95% CI 43.3–98.4%) and 74.1% at three years (95% CI 28.9–93.0%); and for CCMR type 3, 37.5% at one year (95% CI 8.7–67.4%) and 25.0% at two years (95% CI 3.7–55.8%) (**Figures 4d–f**).

**Figure 5:**
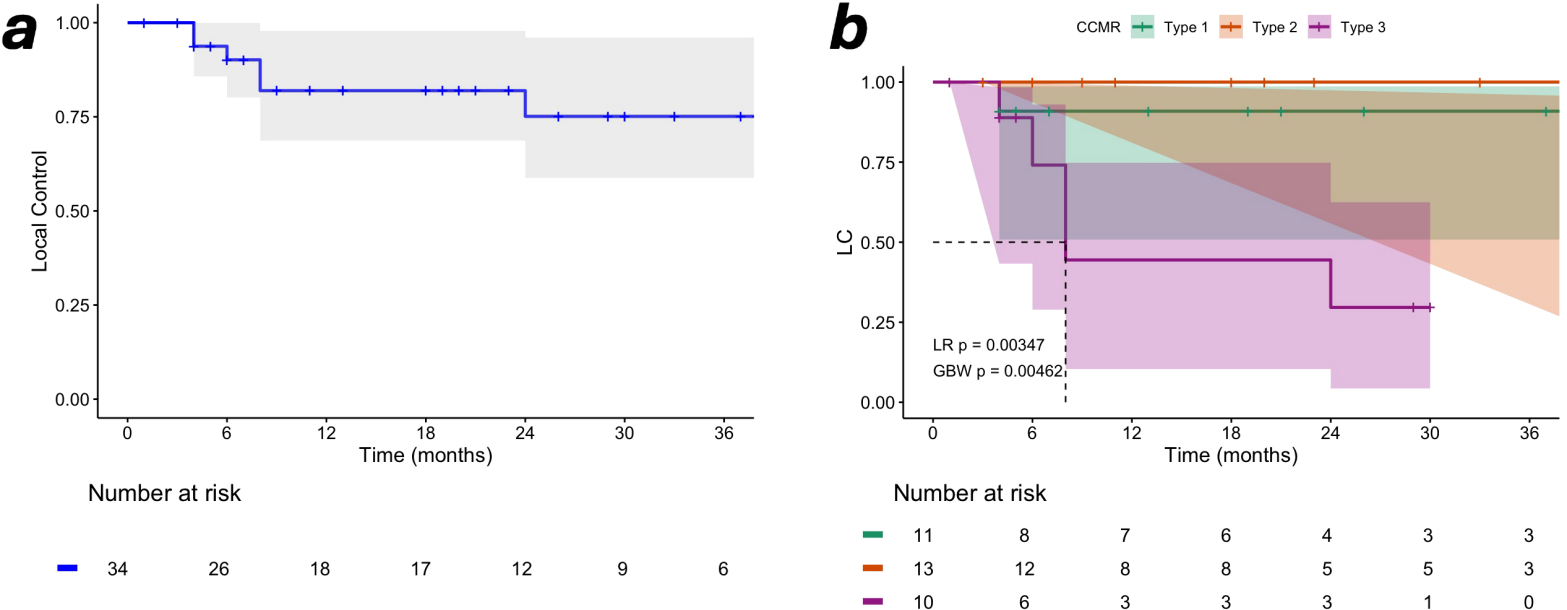
Local control (LC) in the entire cohort **(a)** and by CCMR type **(b)**.

Local control (LC) was analyzed in the entire cohort of 34 patients. One- and three-year LC rates were 81.9% (95% CI, 68.6–97.8%) and 75.1% (95% CI, 58.7–96.1%), respectively (**Figure 5a**).

LC for patients undergoing CCMR type 1 (11/34) was 90.9% at one and three years (95% CI 50.8–98.7%); for CCMR type 2 (13/34), 100% at both one and three years (95% CI not estimable, due to absence of events); and for CCMR type 3 (10/34), 44.4% at one year (95% CI 10.4–74.8%) and 29.6% at two years (95% CI 4.3–62.5%) (**Figure 5b**).

## 4 Discussion

The surgical management of maxillary cancers with deep craniofacial extension remains one of the most demanding challenges in head and neck oncology. In the 6th edition of the AJCC staging system (2002), extension into the ITF/masticator space and pterygoid region was classified as T4 for both MSCs and OCCs; in OCCs, this corresponds to T4b and has traditionally been regarded as unresectable (15). Over the past two decades, however, this binary view has been challenged by evidence showing that selected tumors can be treated with curative intent through anatomically guided compartmental strategies (16).

A major conceptual shift came with Liao (2007), who showed that resectability depends more on infranotch–supranotch anatomy than on the T4b label, with significantly better five-year DFS in infranotch tumors (65% vs. 14%) (14). This principle was later extended to the oromaxillofacial region by Mohiyuddin et al. (2018), who demonstrated that compartmental ITF resections can achieve meaningful local control, particularly in anterior or infranotch disease; notably, close margins and posterior extension, rather than neoadjuvant chemotherapy (NACT), were the main determinants of recurrence (17).

Indian groups subsequently formalized the principles of compartmental masticator space resection. Trivedi et al. (2012) described anatomically coherent, en bloc dissections that removed the pterygoid unit, masseter, pterygoid plates, and mandibular ramus as a single specimen (18). This approach highlighted the difficulty of achieving negative margins at the pterygomaxillary junction and pterygopalatine fissure, regions poorly exposed by traditional geometric resections. These insights introduced the concept of functional margins, anticipating the compartmental philosophy later applied across oral cavity subsites.

Italian groups further consolidated this paradigm, showing that anatomically defined resections of muscular and neurovascular units improve local control compared with traditional wide excisions, especially for tongue and buccal mucosa cancers. Extensions of these principles into the masticator and pterygoid spaces have enabled truly en bloc removal while overcoming the intrinsic limitations of linear resections.

The adoption of minimally invasive, multiportal approaches represented a further advance. Schreiber et al. (2021) combined transnasal endoscopy with transcervical–transmandibular access, reporting posterior negative-margin rates approaching 96%, while improving exposure of the internal maxillary artery and pterygoid plexus. In our series, negative margins were achieved in 79.4% of patients. Our findings reinforce the paradigm proposed by Schreiber et al., demonstrating that the early endoscopic control of the pterygoid region is instrumental in achieving high rates of negative posterior margins, effectively mitigating the risk of local failure (10).

Pillai et al. (2024) then proposed a two-tier classification of compartmental ITF resectionbased on tumor epicenter and mandibular involvement, emphasizing surgical sequences that maximize exposure and early vascular control (19). Reviews by Mattavelli et al. (11) and Accorona et al. (20) reframed resectability as a continuum determined by skull-base proximity, perineural spread, and vascular relationships rather than AJCC stage alone, and highlighted the value of combined endoscopic and open approaches in improving both standardization and radicality (11, 20).

More recently, Mahajan et al. (21) further refined this concept through a four-compartment imaging-based stratification of masticator space and ITF involvement, showing progressively worse outcomes from low ITF to posterior high ITF disease, particularly in cases with perineural extension toward the pterygopalatine corridor (21).

Overall, the available literature suggests that selected T4 maxillary cancers can be resected with curative intent and adequate margins when supported by careful preoperative assessment and compartmental surgical principles. In this context, unresectability should be reserved for frank skull-base invasion and/or encasement of critical vessels.

Our findings align with this evolution. Using a standardized combined compartmental framework, CCMR achieved negative margins in 79.4% of patients, with early survival outcomes broadly consistent with benchmark results from major compartmental and endoscopic-assisted series. The cohort was clinically complex, with more than 40% previously treated, yet margin-negative resection remained feasible even in cases with partial or extensive ITF involvement, supporting the robustness of a compartment-based, multiportal strategy in anatomically high-risk disease.

Although the key anatomical criterion for CCMR selection in this study was deep posterior extension into the ITF, tumor spread to adjacent structures, including the orbital floor, V_2_/V_3_-related foraminal regions, skin, parotid region, or other neighboring compartments, could also influence resectability and the ability to achieve clear margins. These factors were assessed preoperatively, and the extent of resection was tailored accordingly, with intraoperative frozen sections used to evaluate critical margins when appropriate.

The inferior outcomes observed after CCMR type 3 resections are best interpreted as a marker of tumor burden and anatomical complexity rather than as an effect of the template itself, given that type 3 is reserved for the most advanced patterns of spread. The limited number of events and consequent imprecision preclude definitive causal inference; these findings should be considered hypothesis-generating and warrant confirmation in larger series with adjustment for stage and extent-of-disease variables.

Given their distinct biology and consistently poor prognosis in the published literature, mucosal melanoma and NUT carcinoma were excluded from the survival analyses (22, 23). For the case of ameloblastoma, the patient underwent CCMR type 3 because, despite the lesion’s non-malignant classification, it had recurred after prior excision and was progressing towards the skull base with clinically significant symptoms. Because of the extent of disease, occult malignant foci could not be confidently excluded preoperatively. Following definitive histopathological confirmation of ameloblastoma, the case was retained for the surgical technique analysis but excluded from outcome estimation (24).

We acknowledge that the survival outcomes reported in this series should be interpreted in light of several intrinsic limitations, including the small cohort size, histological heterogeneity, and the inclusion of patients with recurrent or persistent disease. In addition, no formal central imaging review was performed. Radiological assessment was conducted locally at each participating center as part of routine multidisciplinary planning and was not standardized across institutions. Consequently, both the classification of ITF involvement and the selection of the surgical template may have been influenced by center-specific interpretative practices.

A further technical limitation is the potential discrepancy between clinical and pathological staging. In extensive resections of the ITF and masticator space, the separate identification, orientation, and labeling of individual masticatory muscles within the surgical specimen may be challenging. This may limit pathological confirmation of the precise anatomical structures considered involved on preoperative imaging.

The present study was not designed to provide definitive survival estimates or to formally stratify cT4b tumors according to infranotch vs. supranotch extension. Rather, its primary objective was to assess the technical feasibility and oncological radicality of a standardized, staged compartmental resection strategy based on the extent of deep posterior tumor spread into the ITF and on the feasibility of achieving clearance of the involved anatomical compartment. This framework was deliberately applied to both OCCs and MSCs, although the definition and clinical implications of cT4b disease differ between these two anatomical sites.

Within this surgical framework, the central anatomical considerations were the preoperative assessment of the deep margin and the feasibility of extending the resection through the ITF to the skull base when required, as in CCMR type 3. Although the infranotch–supranotch distinction remains clinically and prognostically relevant, it was therefore not incorporated as a formal stratification variable in this preliminary cohort.

As larger cohorts become available and the technique undergoes further validation, subgroup analyses of cT4b OCCs according to infranotch or supranotch extension may provide additional prognostic information and help refine patient selection.

The principal contribution of the present study lies instead in the codification of a reproducible surgical strategy for addressing a critical, anatomically complex, and often difficult-to-access region.

By integrating contemporary multiportal access with compartmental surgical principles, our goal is to describe a step-by-step technique that allows for the control of locally advanced disease and supports the achievement of negative margins even in tumors traditionally considered unresectable by current guidelines. The strength of this approach resides in structured imaging-informed preoperative planning and in a codified surgical sequence, which converts the posterior–medial deep limit from a blind maneuver into a visually controlled first step. While larger series with centralized imaging review and longer follow-up are needed to overcome the limitations of survival analysis, the technical codification presented here offers a robust framework for securing the most challenging margins in maxillary oncology.

Collectively, current evidence converges on several key principles. Maxillary cancers with anterior or infranotch extension towards ITF and masticator space can be resected with curative intent, achieving outcomes comparable to T4a disease; compartmental surgery remains the most reliable means of securing negative margins; endoscopic corridors enhance the safety and consistency of access to deep spaces; and surgical margins and nodal status remain the dominant prognostic determinants. Our study builds on this foundation by proposing a scheme for integrating endoscopic and open approaches according to topographic extent. This framework may help standardize practice across centers and supports a shift away from the rigid resectable/unresectable dichotomy toward a more anatomy-driven conception of surgical feasibility in advanced maxillary cancers.

## 5 Conclusion

In this multicenter study, we describe and codify a standardized combined surgical strategy for selected maxillary cancers arising from the oral cavity or maxillary sinus with deep craniofacial extension. Based on fixed anatomical landmarks and preoperative assessment of ITF involvement, CCMR provides an anatomically structured framework for curative-intent, en bloc resection and margin-oriented surgical planning.

Within the limits of a retrospective series, margin-negative resection remained feasible even in cases with partial or extensive ITF involvement, suggesting that masticator space or infratemporal extension should not be considered an absolute marker of unresectability when appropriately selected patients are treated in experienced centers.

Prospective multicenter validation is warranted to clarify the generalizability, reproducibility, and clinical decision-making value of this framework. Further refinements may derive from improved image guidance, patient-specific planning, and systematic integration of reconstructive and rehabilitation pathways, with the aim of translating technical radicality into durable oncologic and functional outcomes.

## Data Availability

The raw data supporting the conclusions of this article will be made available by the authors, without undue reservation.
