# Supplementary material for: The combined compartmental maxillary resection: technique codification and preliminary multicenter series analysis
Source: Front Oncol. 2026 Aug 5;16:1908312. doi: 10.3389/fonc.2026.1908312 (PMC13485794; doi:10.3389/fonc.2026.1908312)
Supplement: Supplementary file 1 [file DataSheet1.docx]

**Supplementary Material**

Exploratory Kaplan–Meier analyses were performed according to disease status at presentation, categorized as primary, persistent, or recurrent disease.

At 3 years, OS was 76.0% (95% CI, 37.9–92.5%) for primary disease, 60.0% (95% CI, 12.6–88.2%) for persistent disease, and 80.0% (95% CI, 20.4–96.9%) for recurrent disease. Three-year DFS was 66.1% (95% CI, 33.9–85.4%), 75.0% (95% CI, 12.8–96.1%), and 40.0% (95% CI, 5.2–75.3%), respectively, whereas 3-year LC was 75.8% (95% CI, 37.7–92.4%), 75.0% (95% CI, 12.8–96.1%), and 66.7% (95% CI, 19.5–90.4%), respectively.

No statistically significant differences were observed for OS (LR p = 0.522; GBW p = 0.494), DFS (LR p = 0.426; GBW p = 0.463), or LC (LR p = 0.734; GBW p = 0.715).

These analyses were exploratory and substantially underpowered because of the small and markedly unbalanced subgroup sizes.

|  |  |  |
| --- | --- | --- |
